# Introducing the Amphibious Mudskipper Goby as a Unique Model to Evaluate Neuro/Endocrine Regulation of Behaviors Mediated by Buccal Sensation and Corticosteroids

**DOI:** 10.3390/ijms21186748

**Published:** 2020-09-14

**Authors:** Yukitoshi Katayama, Kazuhiro Saito, Tatsuya Sakamoto

**Affiliations:** Ushimado Marine Institute, Faculty of Science, Okayama University, Kashino 130-17, Setouchi, Okayama 701-4303, Japan; sc19412@s.okayama-u.ac.jp (K.S.); ryu@uml.okayama-u.ac.jp (T.S.)

**Keywords:** stressors, thirst, angiotensin II, corticosteroids, amphibious fish

## Abstract

Some fish have acquired the ability to breathe air, but these fish can no longer flush their gills effectively when out of water. Hence, they have developed characteristic means for defense against external stressors, including thirst (osmolarity/ions) and toxicity. Amphibious fish, extant air-breathing fish emerged from water, may serve as models to examine physiological responses to these stressors. Some of these fish, including mudskipper gobies such as *Periophthalmodon schlosseri*, *Boleophthalmus boddarti* and our *Periophthalmus modestus*, display distinct adaptational behaviors to these factors compared with fully aquatic fish. In this review, we introduce the mudskipper goby as a unique model to study the behaviors and the neuro/endocrine mechanisms of behavioral responses to the stressors. Our studies have shown that a local sensation of thirst in the buccal cavity—this being induced by dipsogenic hormones—motivates these fish to move to water through a forebrain response. The corticosteroid system, which is responsive to various stressors, also stimulates migration, possibly via the receptors in the brain. We suggest that such fish are an important model to deepen insights into the stress-related neuro/endocrine-behavioral effects.

## 1. Introduction

**Figure 1:**
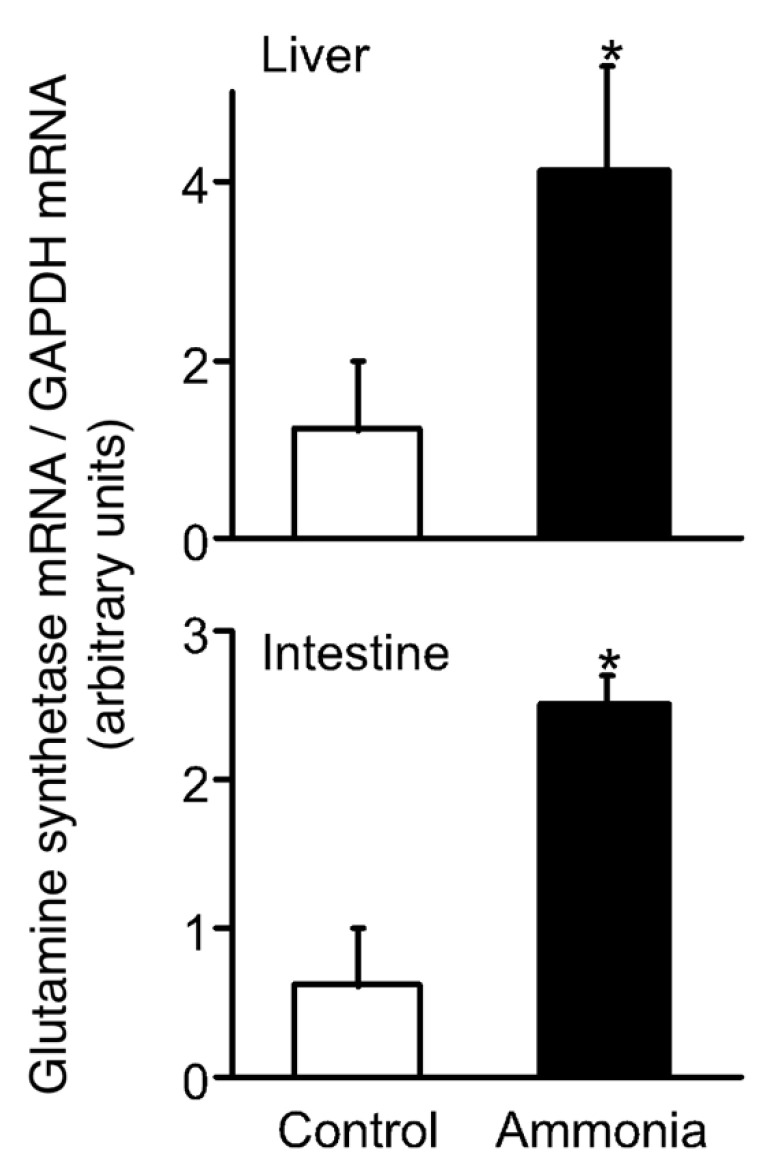
Glutamine synthesis adopted by amphibious fish to defend against ammonia toxicity. Level of glutamine synthetase mRNA in mudskippers exposed for 1 day (*n* = 6). Bars represent the mean ± SEM. * *p* < 0.05 vs control. This strategy involves the detoxification of ammonia to glutamine, whereas the glutamine synthetase activity is generally undetectable or low in nonureosmotic fishes [14,15].

Water–land transition necessitates many important physiological and biochemical adaptations to the terrestrial environment. In particular, fish that routinely emerge from water, such as mudskippers (class Actinopterygii, order Perciformes, family Gobiidae; e.g., *Periophthalmodon schlosseri*, *Boleophthalmus boddarti* and our *Periophthalmus modestus*) commonly found in estuaries of the Indo-Pacific and evolutionarily many other fish taxa [1], cannot effectively flush their body surfaces (primarily gills) due to a lack of water. Hence, these fishes are amphibious and have developed ways to ameliorate terrestrial stress (e.g., endogenous ammonia) during emersion. They are equipped with strategies on land, such as modified nitrogen metabolism (Figure 1), and amphibious behavior to defend against ammonia toxicity, whereas most aquatic animals excrete ammonia in water. Therefore, these fish, as well as ammonia-tolerant nonamphibious fish such as the Gulf toadfish and a gobiid fish (*Mugilogobius abei*) [2,3,4,5,6], may serve as unique models to examine the effects of stressors such as ammonia, but their characteristic adaptational behaviors and neuro/endocrine mechanisms have only been minimally studied [7,8,9,10,11,12,13].

**Figure 2:**
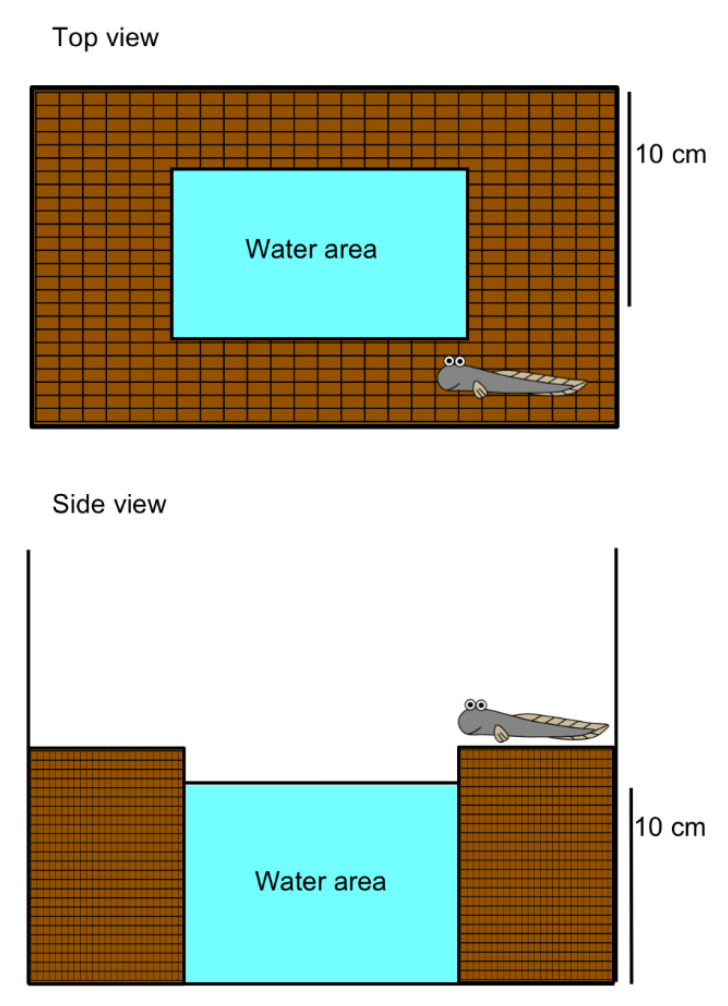
Schematic of the experimental setup used to examine amphibious behavior in mudskippers. The hashed area represents land and is easily accessible to the mudskipper placed in the water area. Treatment with stressors and hormones allows the observation of the period of time in water and the frequency of migration. Plastic mesh on the land area facilitates drainage of water. Water in the tank is constantly aerated [17,24,25,26].

*Periophthalmus modestus* spend a significant amount (70–90%) of time out of water (0–40 parts per thousand) [16,17], as a means of predator avoidance and to forage (Supplementary Video S1), which appears to be ecologically diverse across the families [1]. They have evolved numerous physiological and behavioral traits of locomotion, gas exchange, nitrogen excretion, ionoregulation and osmoregulation associated with amphibious lives [7,8,18,19,20,21,22]. For example, they can store water in the buccal and opercular cavities when they are on land, because their opercula are closed without ventilation of the gills [17]. The aquatic preference and rolling behavior on wet land are also notable for moistening the dorsal skin [23]. The emersion is very easy to score as an individual is either in or out of water, which makes the mudskipper an interesting model to examine the effects of external stressors such as ammonia and dehydration (Figure 2), and may provide novel avenues for investigations of the neuro/endocrine systems, since some of these have recently been analyzed [16].

In this review, we focus on two topics relevant to the neuro/endocrine regulations of stress-related behavioral changes. First, we discuss the behavior motivated by dipsogenic hormones via a buccal local sensation of thirst and external factors such as eating. Then, we introduce the roles of corticosteroids that are responsive to various external stressors [27,28] in the amphibious behavior. We hope that this review will create further interest in the neuro/endocrine regulation of this behavior not only among fish endocrinologists, but also for those working on other animals.

## 2. Migration to Water Motivated by Local Sensation in the Buccal/Opercular Cavity

**Figure 3:**
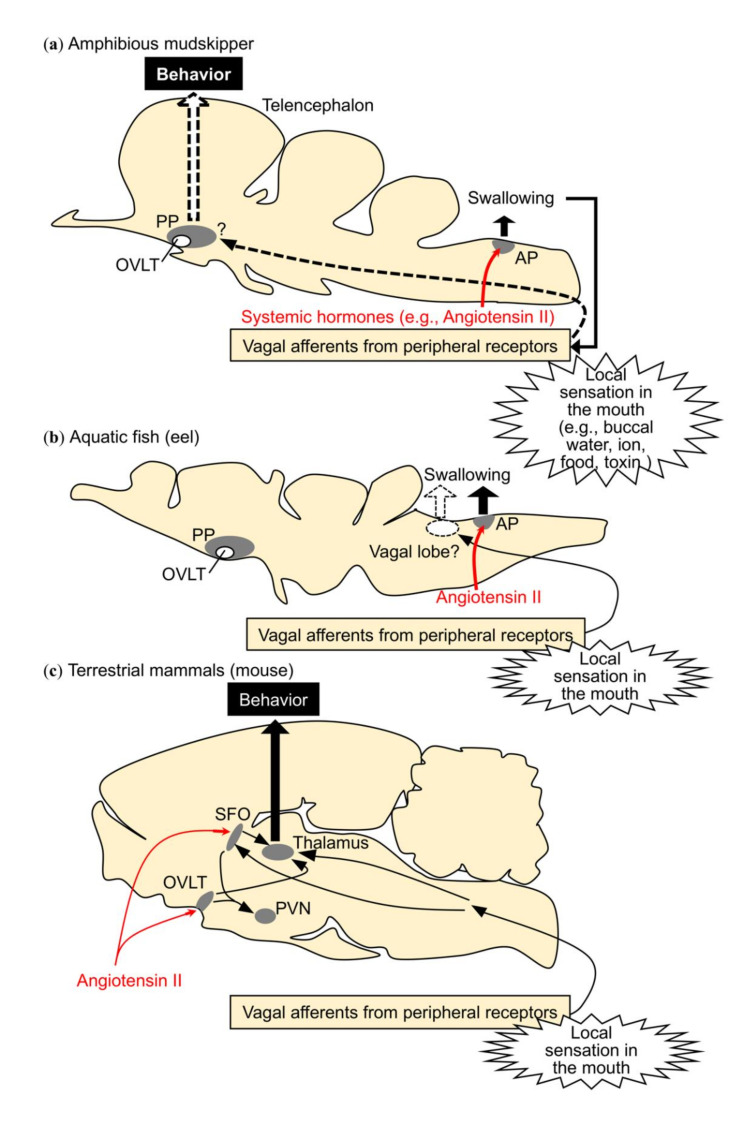
A theoretical model illustrating regulatory mechanisms of drinking behavior in amphibious mudskippers, aquatic fish and terrestrial mammals. The area postrema (AP), organum vasculosum of the lamina terminalis (OVLT) and subfornical organ (SFO) are collectively referred to as the circumventricular organs (CVOs) in the brain. The AP and OVLT have been identified in fish and tetrapods, but the SFO has only been identified in tetrapods. (**a**) Comparatively simple mechanisms in mudskippers. Sensory regions in the buccal cavity of mudskippers monitor the contents (e.g., ion content, water and toxins). Changes in the chemistry of the buccal contents initiates behavioral responses for drinking. Information is processed by afferent vagus/glossopharyngeal nerves, possibly leading to the forebrain initiating migratory behavior to water. The specific region in the forebrain has yet to be identified, but vasotocin nerves in the parvocellular preoptic nucleus (PP) may be involved in the neural basis. Increases in circulatory levels of angiotensin II elicit AP neurons to fire and induce a swallowing response mediated by the medulla oblongata. Loss of buccal water subsequently evokes water-seeking behavioral responses in the forebrain, whereas systemic angiotensin II is perceived by forebrain neurons in mice and motivates a move to water. (**b**) In aquatic fish, the forebrain is not involved in drinking behaviors. Increases in circulatory levels of angiotensin II elicit AP neurons to fire and induce a swallowing response mediated by the medulla oblongata. (**c**) Complex mechanisms in mice. Signals from buccal receptors act through neuronal connections with the forebrain center (e.g., SFO) via visceral afferent neurons in spinal or vagal pathways. These signals are relayed to the thalamus, paraventricular (PVN) and supraoptic (SON) nuclei to promote expression of vasopressin, a mammalian homolog of vasotocin. With the lack of a blood–brain barrier, circulating angiotensin II is perceived by neurons in the SFO and OVLT. The sensation of thirst and subsequent behavioral responses likely involve the activation of the cortex, and these signals may be transmitted to neurons in the medial region of the thalamus. The role of the vagal afferents in regulating behavior relevant to local sensation appears to be conserved among vertebrates. Dashed lines, dashed arrow, and red arrow indicate possible neural signaling, established neural signaling, and hormonal actions, respectively.

On land, mudskippers continually search for water (Supplementary Video S1), in much the same way as tetrapods [16]. Indeed, we recently demonstrated that artificial removal (e.g., by piercing holes in the opercula) of buccal water stored on land by the mudskipper resulted in a strong behavioral preference for a water environment, suggesting the presence of thirst (defined as a conscious sensation of a need for water and a desire to drink, which is followed by a search for water in terrestrial animals such as mammals [29,30]) in fish [17]. Furthermore, administration of the most potent dipsogenic hormone known in many vertebrate species, angiotensin II [31,32,33], resulted in a swallowing action of water held in the buccal cavity of the mudskipper that was regulated by the area postrema (AP) in the hindbrain, as in aquatic fish (Figure 3a,b; [17,24]). Migration of the mudskipper to water following the loss of buccal water by swallowing is indicative of the seeking behavior exhibited by tetrapods in a local thirst (dry mouth) response [16,17]. This behavior is considered to be an anticipatory thirst in mice and is regulated before changes in blood chemistry and/or humoral factors such as angiotensin II [34,35,36].

Buccal water is also used for sucking food when mudskippers eat on land [37,38]. Water volume accumulation is essential for the intraoral transport of food on land, and eating appears to be a potent stimulus for buccal thirst. Indeed, ad libitum eating “naturally” induces immediate migration to water for refilling the buccal/opercular cavity with water (our unpublished data). Many mammalian species similarly drink during meals [34,39,40].

In fully aquatic fish, the anadromous or diadromous migratory behavior induced by local/peripheral sensation is challenging to study; however, work in eels has shown that increases in the buccal Cl^−^ concentration rather than osmoreception or baroreception stimulate a swallowing reflex [41]. This “chloride response” was presumed to prevent the dehydrating effect of life in seawater for hypo-osmotic regulating marine teleosts and was also described as an anticipatory drinking response [41], similar to that described for tetrapods and mudskippers. Additionally, in a basal vertebrate, the river lamprey (*Lampetra fluviatilis*), transfer from seawater to freshwater rapidly decreased the drinking rate without a change in plasma osmolality [42], suggesting that the mechanism of anticipatory drinking by local sensation may be widely distributed among vertebrates. A complex network of the endocrine system also responds to the fluctuation of internal solute concentrations [43]. The integration of hormonal and anticipatory information has recently been shown in mammals [30], but remains to be examined in fish. Recent suggestions of osmosensitive mechanisms in the gill [44,45], as well as in the brain and pituitary [46,47,48], may lead to understanding of the comprehensive mechanisms.

Similarly to osmoregulatory/feeding purposes, these behavioral responses may also protect body surfaces such as gills by flushing with water from external stressors such as ammonia and toxins [23]. Buccal local stimuli are thought to be sensed by afferent fibers of the vagus and/or glossopharyngeal nerves in fish [49]. The input signals for thirst and food consumption are relayed rapidly to neurons in the sensory circumventricular organ (CVO) of the forebrain in mammals (Figure 3c; [35,50,51,52]). The mammalian CVO also monitors blood factors including angiotensin II [31,51,53,54,55,56,57,58,59,60] and orchestrates a motivation for water by engaging the medial thalamic–cortex network [30,61,62]. Given this complex mechanism in mammals, the mudskipper with its simpler brain architecture might be a useful model to investigate neuronal mechanisms of systemic hormone actions separately from local sensation.

## 3. Corticosteroid-Regulated Amphibious Behavior

In vertebrates, corticosteroids released from adrenal or analogous tissue can result in a glucocorticoid or mineralocorticoid response. Tetrapods have evolved responses to two unique hormones: cortisol or corticosterone (species-dependent) primarily regulates energy balance and acts through glucocorticoid receptors (GRs), whereas aldosterone primarily regulates mineral balance and acts through the mineralocorticoid receptor (MR) [63]. In teleosts, aldosterone is unlikely to be present or to act through either receptor [64]; however, circulating 11-deoxycorticosterone (DOC) has been shown to stimulate a MR, but not a GR [64,65,66,67], whereas cortisol appears to act through the GR and MR [64,66,67,68,69,70,71]. In addition to their role in regulating metabolic balance, including the important osmoregulatory role of GRs, GRs and MRs are involved in regulating responses to stress in the brain [28,72,73,74]. For example, the mRNA expression patterns of teleost MR are relatively modest in organs associated with ionoregulation, such as the gills, but are considerably higher in the brain and eyes of most teleosts examined to date (e.g.,: [67,75,76,77]). This suggests that MR signaling may be involved in facilitating behavioral responses to external stimuli.

**Figure 4:**
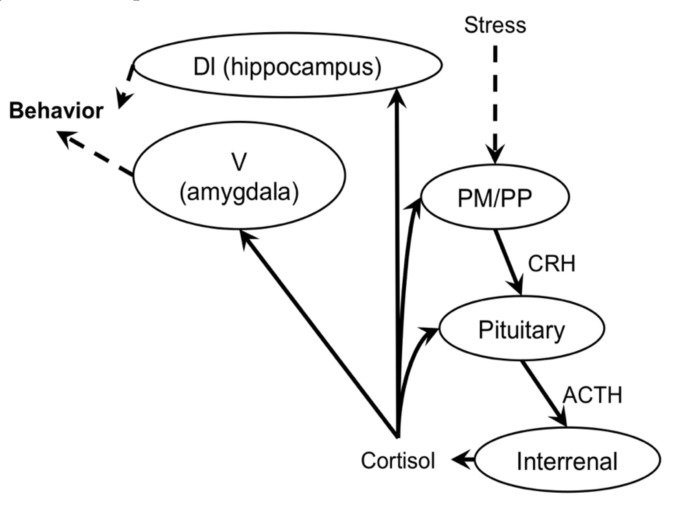
Hypothalamus–pituitary–interrenal axis activation by stress and behavioral modification in fish. The magnocellular preoptic nucleus (PM) and parvocellular preoptic nucleus (PP) of the hypothalamus are stimulated to express corticotropin-releasing hormone (CRH) and possibly vasotocin/isotocin following exposure to stressful stimuli. Binding of CRH causes release of adrenalcorticotrophic hormone (ACTH) from the pituitary gland, which then induces release of cortisol from the interrenal gland. Cortisol binds to GR/MRs in the commissural and subcommissural nuclei of the telencephalon (V; putative fish homologue to the mammalian amygdala) and the ventral parts of the lateral zone of the dorsal telencephalon (DI; putative fish homologue of the mammalian hippocampus) to modulate behaviors such as the aquatic preference of mudskippers. In the central nervous system, cortisol also functions at the level of the pituitary gland and the PP/PM. Dashed lines indicate possible signaling.

Mudskippers migrate into water when treated with DOC and cortisol [25]. Cortisol may act as an endogenous ligand for brain MRs as well as GRs to stimulate this migration naturally, because plasma cortisol, rather than DOC, is increased in dehydrated mudskippers under terrestrial conditions (Figure 4; [22]) and the cortisol-stimulated behavior is not completely inhibited by a specific GR blocker, RU-486. Thus, the aquatic preference of mudskippers, induced by cortisol-brain MR/GR signaling, appears to be a stress response. There are limited data available on such corticosterone responses in amphibians [78].

In addition to being key components of the stress axis, GR is expressed ubiquitously and MR is expressed in several important areas (Table 1) in the fish central nervous system, with likely correspondence to regions of expression in other vertebrates [4,66,77,79,80,81,82,83]. MR is highly expressed in telencephalic regions, which include the ventral parts of the lateral zone of the dorsal telencephalon (putative fish homologue of the mammalian hippocampus [84]), and commissural and subcommissural nuclei of the telencephalon (putative fish homologue of the mammalian amygdala [84]). In the diencephalon, several hypothalamic nuclei and the glomerulus complex of the thalamus exhibit MR expression, as do the mesencephalic tegmentum and granular layer of the optic tectum. MR is expressed markedly in some regions of the cerebellum and eyes. These findings suggest that GRs/MRs are critical for many physiological and behavioral responses, such as the regulation of salt intake, mood, appetite, exploratory behavior and visual responses [77,85,86,87]. Indeed, zebrafish and medaka models with constitutive GR or MR knockout fail to integrate these brain behavior and visual responses as well as regulate the stress axis, although they can grow and osmoregulate [77,80,88,89,90,91,92,93,94,95,96,97]. The simple aquatic preference of mudskippers may also reflect these behaviors. Furthermore, most GR/MR-expressing PM and PP nuclei produce vasotocin and its paralogue, isotocin [82], and the cortisol-GR system regulates the expressions of vasotocin and isotocin [98]. These neuropeptides also promote migration to water in mudskippers, similarly to angiotensin II [26]. In the mammalian brain, neurons in CVOs such as the SFO and AP also express MR [99]. “Crosstalk” among these systems in the brain may be affected by stress. Regardless, the dual-label brain regions for corticosteroid receptors and Fos in mudskippers after the stress-induced aquatic preference suggest their targets.

## 4. Summary and Perspectives

Extant amphibious fish may offer an opportunity to detect stress-related behavior and to examine its neuro/endocrine regulation. These fish can emerge from water and make an excursion onto land, as a means of predator avoidance and to forage. During this emersion, they would have difficulties flushing their gills effectively. Hence, they are equipped with behavioral strategies to defend against terrestrial stressors such as dehydration and ammonia. Assessment of the distinct amphibious behavior of mudskippers as simple methods for detection of stress-related behavioral outcomes is possible compared with fully aquatic fish, and some neuroendocrine regulations of this behaviors have been described here. These studies have demonstrated the potential effects on behavior regulated by dipsogenic hormones via local sensation in the buccal cavity and by the corticosteroid system. Further analyses of natriuretic peptides and gastrin-releasing peptide in mudskippers may elucidate the itch sensation by the dry dorsal skin moistened by aquatic preference or rolling behavior on wet land, and the unknown evolution of the itch sensation in vertebrates. These peptides are currently known as key molecules to transmit the itch sensation to the central nervous system in rodents [100,101,102,103,104]. The factors such as stressors driving many amphibious fish to water have been reviewed, and these factors can also be triggered in cyprinodontiforms, including in model species (e.g., *Kryptolebias marmoratus*) [105,106]. The mode of action can be assessed through neurohistological methods of labeling of Fos and neuro/endocrine transmitter systems in the less complicated fish brain [16], since these species have sequenced genomes [7,107,108,109].

## Figures and Tables

**Table 1:** Distribution of mineralocorticoid receptor (MR) mRNA in the teleost fish brain.

Brain Region	MR
Stress axis Forebrain pallial area Corticotrophin-releasing hormone cells in preoptic nucleus Adrenocorticotropic hormone-containing cells in pituitary pars distalis	++ + +
Ventral parts of the lateral zone of dorsal telencephalon (presumed hippocampus) Commissural and subcommissural nuclei of telencephalon (presumed amygdala) Several hypothalamic nuclei Glomerulus complex of thalamus Mesencephalic tegmentum Granular layer of optic tectum Cerebellum	++ ++ ~ ++ + ++ +++ +++

For details, see Sakamoto et al. [99] and Sakamoto and Sakamoto [80].

## Supplementary Materials

Supplementary materials can be found at https://www.mdpi.com/1422-0067/21/18/6748/s1. Supplementary Video S1 (*Periophthalmus modestus*: Amphibious behavior).
